# Human papillomavirus, human immunodeficiency virus, and esophageal squamous cell carcinoma in South Africa: a study of prevalence, co-infection, and risk factors

**DOI:** 10.3389/fonc.2025.1634565

**Published:** 2025-08-18

**Authors:** Sikhumbuzo Z. Mbatha, Tania Maphosho, Dakalo Ramali, Botle Damane, Portia Nevhungoni, Michelle Mc-Cabe, Benny Mosoane, Rodney Hull, Zodwa Dlamini

**Affiliations:** ^1^ Department of Surgery, Steve Biko Academic Hospital, University of Pretoria, Hatfield, Pretoria, South Africa; ^2^ South African Medical Research Council, Biostatistics Research Unit, Pretoria, South Africa; ^3^ Department of Anatomical Pathology, National Health Laboratory Services: Tshwane Academic Division, Faculty of Health Sciences, University of Pretoria, Hatfield, Pretoria, South Africa; ^4^ SAMRC Precision Oncology Research Unit (PORU), DSI/NRF SARChI Chair in Precision Oncology and Cancer Prevention (POCP), Pan African Cancer Research Institute (PACRI), University of Pretoria, Hatfield, Pretoria, South Africa; ^5^ Wolfson Wohl Cancer Research Centre, School of Cancer Sciences, University of Glasgow, Bearsden, Glasgow, United Kingdom

**Keywords:** esophageal squamous cell carcinoma (ESCC), human papillomavirus (HPV), human immunodeficiency virus (HIV), South Africa, HPV genotyping, oncogenic viruses, esophageal carcinogenesis, viral co-infection

## Abstract

**Background:**

Esophageal squamous cell carcinoma (ESCC) is a serious public health concern in South Africa, ranking among the most lethal malignancies. It has known risk factors including human papillomavirus (HPV). HPV is strongly linked to squamous cell cancers (i.e., cervix, anus, and oropharynx) with human immunodeficiency virus (HIV) shown to increase susceptibility to HPV-related malignancies. The extent to which co-infection with these two viruses contribute to ESCC in South African populations is unclear. This study aimed to determine the prevalence of HPV and HIV in ESCC patients.

**Methods:**

A total of 78 ESCC patients were prospectively recruited between January 2022 and December 2024 at Steve Biko Academic Hospital, Pretoria, South Africa. Participants were assessed for HIV, and tumors biopsied by endoscopy. DNA was extracted from formalin-fixed paraffin-embedded (FFPE) tissue specimens and HPV detection and genotyping were performed. Statistical analyses were conducted using Stata 18, with chi-square tests and logistic regression applied to assess associations, using a significance threshold of p ≤ 0.05.

**Results and discussion:**

The study population was predominantly Black Africans (96%), 55% male and 45% female, and aged 34–86 years. HIV infection was present in 42.3% (n=33) of patients. High-risk HPV DNA was detected in 56.4% (n=44) of ESCC cases, with high-risk subtypes HPV16 and HPV18 being the most prevalent, found in 68% and 41% of HPV-positive cases, respectively. Co-infection with both HIV and HPV was observed in 23.1% (n=18) of patients. However, statistical analyses showed no significant association between HIV and HPV status in ESCC patients (p = 0.78). However, a trend towards correlation was noted between HIV status and HPV18 positivity (adjusted p = 0.051).

**Conclusion:**

While no direct association between HIV and HPV in ESCC was found, the high prevalence of high-risk HPV, particularly HPV16 and HPV18, highlights the need for further research. Given South Africa’s burden of HIV and HPV, larger multicenter studies are essential to better understand viral contributions to esophageal carcinogenesis.

## 1 Introduction

GLOBOCAN 2022 statistics show that esophageal cancer is the seventh leading cause of cancer-related mortalities worldwide, despite being the eleventh most prevalent form of cancer. There are two main histological subtypes: esophageal squamous cell carcinoma (ESCC) and adenocarcinoma, which exhibit distinct regional distributions. Adenocarcinoma is more common in North America and Western Europe, while ESCC predominates in Africa and Asia. (1). ESCC accounts for approximately 85% of global esophageal cancer cases, while adenocarcinoma accounts for only 14%. The highest incidence rates of ESCC were observed in East Asia, followed by Eastern and Southern Africa (2). In 2021, the Global Burden of Disease study identified East Asia, Southern Sub-Saharan Africa, and Eastern Sub-Saharan Africa as the regions with the highest esophageal cancer age-standardize incidence rates of 14.83, 11.01, and 10.93, respectively. Additionally, these regions also recorded the highest age-standardized mortality rates with East Asia at 13.91, Eastern Sub-Saharan Africa at 11.74, and Southern Sub-Saharan Africa at 11.69 (3).

South Africa (SA) has experienced a steady rise in the incidence of esophageal carcinoma over the past decades. This has been attributed to alterations in diet, lifestyle and increasing exposure to carcinogens; and as a result, the country currently faces a particularly high burden of ESCC, with disproportionately high incidence rates in the Eastern Cape, KwaZulu-Natal, and parts of the Gauteng province (4, 5). This cancer is often discovered at an advanced stage, leading to poor prognosis. A South African statistics report for year 2019 showed that despite esophageal cancer being the eight most common cancer in the country, it was responsible for a third of cancer deaths among males (6).

The established risk factors for ESCC include genetic susceptibility, dietary deficiencies (such as low intake of fruits and vegetables), chronic esophageal irritation (e.g., from hot beverages), metabolic disorders, smoking, alcohol consumption, and viral infections (7, 8). Among viral risk factors, human papillomavirus (HPV) infection, particularly high-risk subtypes HPV16 and HPV18, have been implicated in esophageal carcinogenesis (7). HPV is well-established as a carcinogen in cervical, anogenital, and oropharyngeal cancers, and similar mechanisms, — viral oncoproteins E6 and E7 inactivating tumor suppressor proteins p53 and pRb,− are believed to contribute to ESCC development (9, 10). However, while HPV has been detected in high-incidence ESCC regions, its prevalence remains low in regions with lower ESCC incidence (11).

South Africa has one of the highest HIV prevalence rates in the world, with approximately 12.7% (8 million people) living with HIV in 2024 (12), and is credited with the biggest anti-retroviral therapy (ART) program globally (13). The HIV-related immune suppression is thought to increase susceptibility to oncogenic infections such as HPV and hepatitis viruses, reduces immune clearance of malignant cells and accelerates carcinogenesis (14). While the widespread adoption of ART has improved survival rates for people living with HIV (PLWHIV), it has also led to a rise in non-AIDS defining cancers (NADCs), particularly the virally associated malignancies such as HPV-driven cancers, including ESCC (15, 16).

In South Africa, the relationship between HIV, HPV, and ESCC remains insufficiently explored. Given the country’s burden of HIV, HPV and ESCC, further research is needed to determine whether HIV-associated immunosuppression enhances the oncogenic potential of HPV in the esophagus, as it does in cervical cancer. This study seeks to describe the prevalence of these two viral infections in a South African ESCC population by analyzing HPV prevalence and genotype in tumor tissue, quantifying the proportion of viral co-infections, and assessing if HIV positivity is associated with greater likelihood of also detecting HPV in tumors.

## 2 Materials and methods

### 2.1 Study setting

This study was a cross-sectional study conducted at Steve Biko Academic Hospital (SBAH), a tertiary referral center affiliated with the University of Pretoria. SBAH is located in Pretoria, Gauteng, South Africa and is one of three academic hospitals in the province; it serves patients from the central and eastern Pretoria region and Mpumalanga province. Ethical approval was obtained from the University of Pretoria Research Ethics Committee (Ethics Reference No.: 296/2021) and the Gauteng Health Department (NHRD Ref No.: GP_202107_062).

### 2.2 Patient recruitment and sample collection

**Figure 1:**
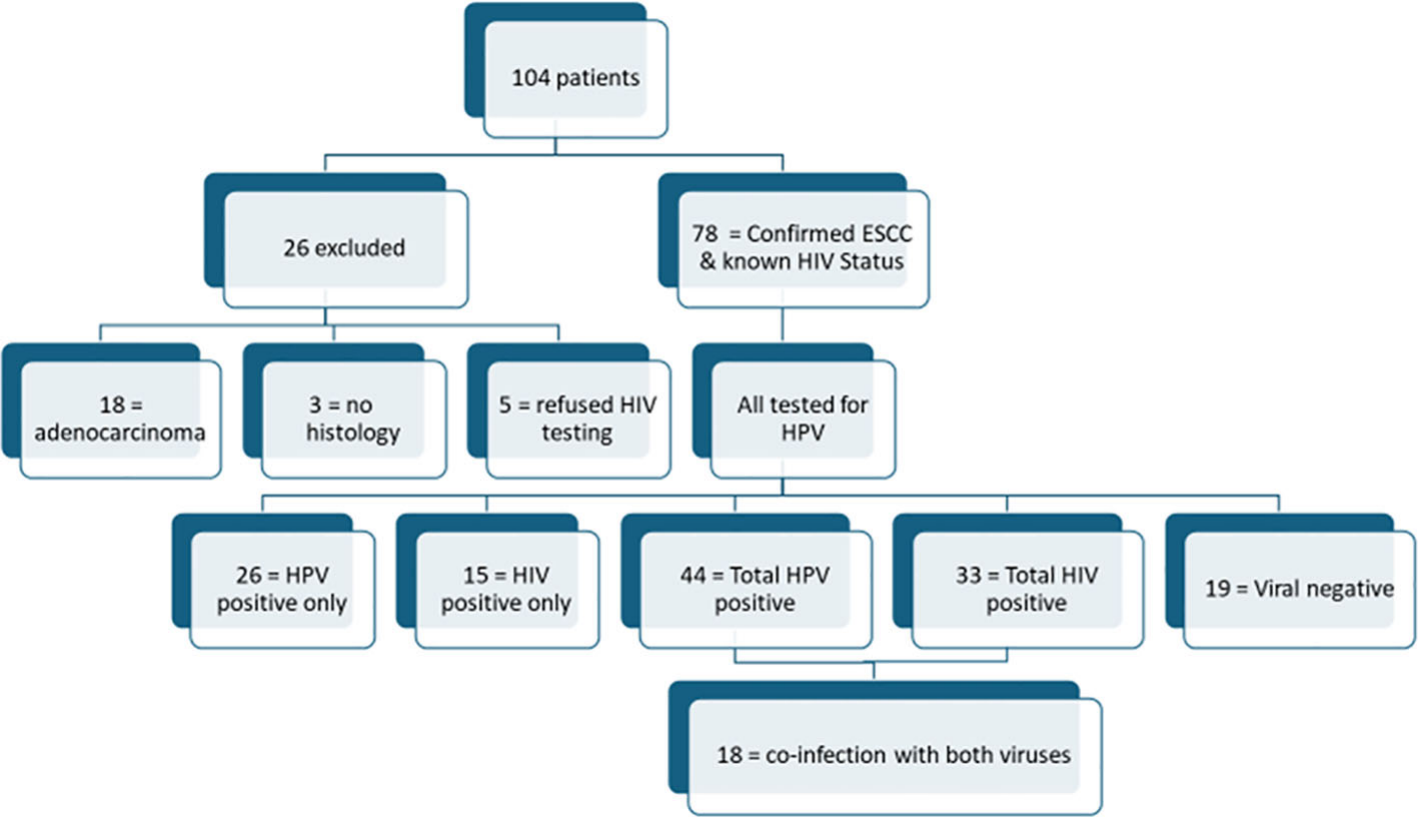
Flow diagram of patient breakdown: All the 78 included patients were tested for HPV infections. While a significant number of individuals were infected with either HIV or HPV individually, only 23% (n=18) of patients had co-infection with both viruses. For the three patients with “no histology”, their specimens and results could not be traced on the NHLS results database and patients were lost to follow-up, and as such repeat biopsied could not be done.

A total of 104 consecutive patients presenting with clinical characteristics suggestive of esophageal cancer at the SBAH Department of Surgery were prospectively recruited between January 2022 and December 15, 2024. Patients underwent a questionnaire-based medical history taking including inquiries about their HIV status; and after obtaining the necessary informed consent, a blood sample was taken for HIV testing using the enzyme-linked immunosorbent assay (ELISA) from those who were not already taking HIV antiretroviral therapy (ART). Each patient underwent esophagogastroscopy, during which multiple random biopsies were collected from tumors using standard disposable, single-use endoscopic biopsy forceps. The biopsy specimens were immediately fixed in 10% buffered formalin and sent for histopathological evaluation by the National Health Laboratory Services (NHLS), Tshwane Pathology Academic Division. Of the 104 recruited patients, 78 patients met the inclusion criteria and were included in the final study cohort and twenty-six (26) patients were excluded. The inclusion criteria were histological confirmation of esophageal squamous cell carcinoma (ESCC) and objectively confirmed HIV status (either already on ART or confirmed via ELISA). Excluded patients comprised of eighteen (n=18) adenocarcinomas on histology, five (n=5) refused HIV testing and three (n=3) were excluded due to missing biopsy results. **Figure 1** provides a flow diagram depicting patient selection.

### 2.3 Conventional histology

For conventional histological, tissue processing was initiated at 40°C using a 10% buffered formaldehyde solution, followed by exposure to graded alcohol solutions. A clearing agent was applied in a 50:50 ratio with absolute alcohol, followed by a two-step clearing process. The tissue was then infiltrated with molten paraffin wax in three steps at 60°C. Automated processing programs exposed tissue samples to different solutions at 15- to 45-minute intervals, depending on fragment size. Paraffin-embedded sections of 4-micron thickness were obtained using steel-blade microtomy. Hematoxylin and eosin staining was performed, with intermittent nuclear bluing using Scot’s water and subsequent rinsing. This was followed by exposure to graded alcohol solutions, clearing, and mounting.

### 2.4 DNA extraction

Following histological confirmation of ESCC, FFPE tissue blocks were retrieved. The first batch of 49 FFPE blocks was retrieved and processed in April 2024 and the final batch (n=29) was retrieved and processed in December 2024. For sectioning, sections were cut into scrolls at 8μm thickness. To minimize cross-contamination sections were cut from each tissue block using a new clean and sterile blade on a microtome for each sample. The new blade and cutting surface area were cleaned before and after cutting with 10% sodium hypochlorite followed by 70% ethanol and DNAase away solution. The first 2–3 sections cut from each sample block were discarded. The scrolls were then placed in sterile 2ml microcentrifuge tubes. DNA was extracted from these sections utilizing the QIAamp DNA FFPE Tissue Kit (Qiagen, Cat. No. 56404, Hilden, Germany) following the manufacturer’s protocol, with slight modifications. Briefly, FFPE sections were deparaffinated by adding 1 ml of xylene, vortexing for 10 seconds, and centrifuging at 15000g for 2 minutes at room temperature. The supernatant was carefully removed without disturbing the pellet. Residual xylene was washed off with 1 ml of absolute ethanol, followed by vortexing and centrifugation at full speed for 2 minutes. Ethanol was then evaporated by incubation at 37°C. The pellet was resuspended in 180μl of Buffer ATL (animal tissues lysis buffer), mixed with 20μl of proteinase K, and incubated at 56°C overnight for complete tissue digestion.

After a 30-second centrifugation, 200μl of Buffer AL (lysis buffer) and 200μl of 100% ethanol were added sequentially with vortexing. The lysate was transferred onto a QIAamp MiniElute column in a 2 ml collection tube and centrifuged at 8000g for 1 minute. The column was washed sequentially with 500μl of Buffer AW1 (wash buffer 1) and Buffer AW2 (wash buffer 2), each followed by centrifugation at 8000g for 1 minute. A final centrifugation was performed at 15,000 g for 3 minutes. The column was then placed in a clean microcentrifuge tube, and DNA was eluted by adding 50μl of Buffer ATE (aqueous elution buffer), incubating at room temperature for 1 minute, and centrifuging at 15,000g for 1 minute. For optimal DNA recovery, a 5-minute extended incubation before elution was recommended. The extracted DNA was stored at -20°C for downstream applications such as quantitative PCR (qPCR).

### 2.5 Quantification and HPV genotyping by real-time PCR

Prior to qPCR analysis, DNA concentration was measured using the BioDrop μlite+ spectrophotometer (Harvard Bioscience Inc. Holliston, Massachusetts, USA) and adjusted as per protocol for accurate amplification. HPV detection and genotyping were performed using the Sacace HPV 14 Screening & 16,18,45 Typing Real-TM Quant PCR Kit (Sacace Biotechnologies, Como, Italy). Each 25 µL PCR reaction contained 40ng/μl of genomic DNA, 1x PCR-mix-1-FRT HPV 14, PCR-mix, 0.5μl of TaqF DNA Polymerase.

Control reactions included 10μl of QS HPV C1 and QS HPV C2 for HPV 16, 18, and 45 genotyping, along with 10μl of the Negative Control reagent.

PCR was performed with the following conditions: initial denaturation at 95°C for 15 minutes, followed by 45 cycles of denaturation at 95°C for 30 seconds, annealing/extension at 60°C for 25 seconds, with fluorescence detection.

Fluorescence was recorded at 60°C using the Joe (Yellow)/HEX, Fam (Green), Rox (Orange), and Cy5 (Red) channels. Standard curves were generated using quantitative standards, and HPV DNA levels in clinical samples were determined by comparing fluorescence intensity to the standard curve.

### 2.6 Statistical analysis

Statistical analyses were conducted using Stata 18 (StataCorp LLC, College Station, Texas, USA). The relationships between ESCC, HIV infection, HPV infection, and co-infection with HIV and HPV were evaluated, along with demographic variables using chi-square tests or Fisher’s exact tests when expected cell counts were low. The Benjamini-Hochberg procedure was applied to adjust p-values for multiple testing with a false discovery rate (FDR) set at 5%. Variables with adjusted p-values <0.05 were considered statistically significant. Univariate logistic regression and Firth’s penalized logistic regression were performed to evaluate the association between HPV or HIV infection status and selected risk factors, adjusting for potential confounders where appropriate, while accounting for the small sample size and low variability. Missing data were handled using complete case analysis for all regression models. Descriptive analyses included all available data per variable.

## 3 Results

### 3.1 Demographics

**Table 1** presents the demographic, lifestyle, and clinical characteristics of the study cohort. Seventy-eight patients (N=78) diagnosed with ESCC and with known HIV status were included in the final analysis. The majority (96.2%, n=75) were of black African descent. Males comprised 55.1% (n=43) of the cohort, while females accounted for 44.9% (n=35). The patients’ ages ranged from 34 to 86 years. The age distribution showed that 87% of patients were over 50 years old, with the remaining 13% aged 50 or younger. In terms of HIV status, 42.3% of patients were HIV positive, whereas 57.7% were HIV negative. HPV infection was found in 56.4% of patients; 38.5% of HPV-positive individuals were infected with HPV16, and 23.1% with HPV18. Dietary habits revealed that 87.2% of patients consumed maize products as a staple food. Only a small proportion of patients reported eating rice (5.1%) or pasta (1.4%). Approximately 71.8% of patients consumed hot beverages on a regular basis, whereas 65.4% reported eating vegetables frequently. In terms of comorbidities, 23 patients had at least one comorbid condition, with hypertension being the most common accounting for 69.6% of all the co-morbidities. A history of cancer in the family was reported by 11.5% of the patients. Alcohol consumption was reported by 44.2% of patients, while 41.0% reported smoking. In one patient the history of alcohol use or comorbidities was not captured on questionnaire. **Table 1** below presents the demographic characteristics of the patient cohort.

**Table 1:** Demographic and clinical characteristics of ESCC patients.

Variable	n (%)	Total
Gender		78
Male	43 (55.1)	
Female	35 (44.9)	
Race		78
African	75 (96.2)	
Caucasian	2 (2.6)	
Mixed	1 (1.3)	
Age, Mean (SD), (Range)	61.6 (10.5); (34 - 89)*	78
Age group		78
≤50	10 (12.8)	
>50	68 (87.2)	
HIV status		78
Negative	45 (57.7)	
Positive	33 (42.3)	
HPV		78
Negative	34 (43.6)	
Positive	44 (56.4)	
HPV16		78
No	14 (17.9)	
Yes	30 (38.5)	
HPV Not Detected	34 (43.6)	
HPV18		78
No	26 (33.3)	
Yes	18 (23.1)	
HPV Not Detected	34 (43.6)	
Staple Diet		73*
Maize based	68 (93.2)	
Pasta	1 (1.4)	
Rice	4 (5.5)	
Diet including frequent consumption of vegetables		78
No	27 (34.6)	
Yes	51 (65.4)	
Frequent consumption of hot drinks		78
No	22 (28.2)	
Yes	56 (71.8)	
Co-morbidities		77*
No	54 (70.1)	
Yes	23 (29.9)	
Type of Cor-morbidities		23^B^
Arthritis	2 (8.7)	
Asthmatic	1 (4.3)	
HPT	16 (69.6)	
DM	3(13.0)	
Tuberculosis on Rx.	1 (4.3)	
Family history of cancer		78
No	69 (88.5)	
Yes	9 (11.5)	
Alcohol and tobacco consumption		
Alcohol consumption		77*
No	43 (55.8)	
Yes	34 (44.2)	
Smoking		78
No	46 (59.0)	
Yes	32 (41.0)	
Both alcohol and smoking		77*
No	49 (63.6)	
Yes	28 (36.4)	

The descriptive demographic and clinical characteristics of the 78 patients with esophageal squamous cell carcinoma (ESCC) included in the study.

*Data were missing for one patient regarding history of alcohol use and comorbidity status; and data missing for five patients regarding staple diet. ^A^44 indicates the total number of HPV positive patients. ^B^23 is the total number of patients with known co-morbidity status.

### 3.2 Viral infections in ESCC

Approximately 42.3% of patients were infected with HIV, while HPV infection was detected in 56.4% (n=44). The most prevalent HPV strains in this cohort were HPV16 and HPV18, accounting for 68,2% and 40,9% of HPV-positive cases, respectively. Co-infection with both HIV and HPV was identified in 18 patients. Among the 44 individuals who tested positive for HPV, 7 (15,9%) were co-infected with both HPV16 and HPV18, while 37 (84,1%) tested negative for concurrent HPV16 and HPV18 infection. HPV45 was detected in four samples, while two samples tested positive for multiple strains, including HPV31, 33, 35, 39, 51, 52, 56, 58, 59, 66, and 68.

The majority of the group did not have co-infections with HIV and HPV, neither did they have any significant co-existence of the two most prevalent HPV strains, namely HPV 16 and 18. Out of the total cohort of 78 patients only 23.1% were found to be simultaneously infected with both viruses, whilst only 15.9% of HPV positive group had both HPV16 and 18.

A chi-square test was used to assess the relationship between HIV status and HPV infection among 78 patients with esophageal squamous cell carcinoma (ESCC). **Table 2** shows that there was no evidence of association between HIV and HPV status (p = 0.776). Among HIV-positive patients, 18 (40.9%) were infected with HPV, with an anticipated frequency of 18.6, giving a Chi-Squared contribution of 0.02, whereas, 15 HIV-positive patients (44.1%) were HPV-negative, with an expected frequency of 14.4 and a similar contribution of 0.02. Furthermore, 26 (59.1%) of the HIV-negative people were HPV-positive, with an expected frequency of 25.4, contributing 0.01. Also, 19 HIV-negative patients (55.9%) were not infected with HPV, which is commensurate with the expected frequency of 19.6 and contributed 0.02 to the Chi-Squared statistic. Overall, the chi-squared value was 0.08 with 1 degree of freedom, and the effect size (Cramér’s V) was 0.03.

**Table 2:** Chi-square analysis of the association between HIV status and HPV infection among OSCC patients.

HIV status	HPV infection	Observed frequency	Expected frequency	χ² contribution	P-value (χ² Test)
Positive	Infected	18 (40.9%)	18.6	0.02	
Positive	Not Infected	15 (44.1%)	14.4	0.02	
Negative	Infected	26 (59.1%)	25.4	0.01	
Negative	Not Infected	19 (55.9%)	19.6	0.02	
Total		N=78	N=78	0.07	0.776
Chi-Squared Statistic (χ²):			0.08		
Degrees of Freedom (df):			1		
Effect Size (Cramér’s V):			0.03		

We conducted additional statistical analyses to examine the associations between individual viruses and various factors in ESCC patients (**Table 3**). Chi-square analysis was performed to assess the relationship between HIV status and demographic, lifestyle, and clinical factors, and the Benjamini-Hochberg procedure was applied to control for false discovery rate at 5%. After adjusting for multiple comparisons, only smoking remained significantly associated with HIV status (adjusted p = 0.045). Specifically, 60.6% (20/33) of HIV-positive patients reported smoking as opposed to 26.7% (12/45) of HIV-negative patients.

**Table 3:** Chi-square analysis of demographic, lifestyle, and clinical factors by HIV status in OSCC patients.

Variable	HIV status		Total	Raw p -value	BH-adjusted p-value (FDR 5%)
	Negative	Positive			
	N=45	N=33	N=78		
Gender, n (%)				0.405	0.603
Male	23 (51)	20 (61)	43 (55)		
Female	22 (49)	13 (39)	35 (45)		
Race, n (%)				1.000*	1.000
African	43 (96)	32 (97)	75 (96)		
Caucasian	1 (2)	1 (3)	2 (3)		
Mixed	1 (2)	0 (0)	1 (1)		
Age group, n (%)				0.015*	0.051
≤50	2 (4)	8 (24)	10 (13)		
>50	43 (96)	25 (76)	68 (87)		
HPVstatus, n (%)				0.776	0.895
Negative	19 (42)	15 (45)	34 (44)		
Positive	26 (58)	18 (55)	44 (56)		
HPV16, n (%)				0.317	0.528
HPV16 Negative	6 (13)	8 (24)	14 (18)		
HPV16 Positive	20 (44)	10 (30)	30 (38)		
HPV Not Detected	19 (42)	15 (45)	34 (44)		
HPV18, n (%)				0.015	0.051
HPV18 Negative	20 (44)	6 (18)	26 (33)		
HPV18 Positive	6 (13)	12 (36)	18 (23)		
HPV Not Detected	19 (42)	15 (45)	34 (44)		
Staple diet, n (%)				0.461*	0.603
maize	37 (92)	31 (94)	68 (93)		
pasta	0 (0)	1 (3)	1 (1)		
rice	3 (8)	1 (3)	4 (5)		
Vegetables (often), n (%)				0.099	0.221
No	19 (42)	8 (24)	27 (35)		
Yes	26 (58)	25 (76)	51 (65)		
Hotdrinks, n (%)				0.875	0.938
No	13 (29)	9 (27)	22 (28)		
Yes	32 (71)	24 (73)	56 (72)		
Cor-morbidities, n (%)				0.151	0.283
No	28 (64)	26 (79)	54 (70)		
Yes	16 (36)	7 (21)	23 (30)		
Cancer family history, n (%)				0.482*	0.603
No	41 (91)	28 (85)	69 (88)		
Yes	4 (9)	5 (15)	9 (12)		
Alcohol, n (%)				0.012	0.051
No	30 (68)	13 (39)	43 (56)		
Yes	14 (32)	20 (61)	34 (44)		
Smoking, n (%)				0.003	0.045
No	33 (73)	13 (39)	46 (59)		
Yes	12 (27)	20 (61)	32 (41)		
Both alcohol and smoking, n (%)				0.017	0.051
No	33 (75)	16 (48)	49 (64)		
Yes	11 (25)	17 (52)	28 (36)		
Both HPV16 and HPV18 Positive, n (%)				0.103*	0.221
No	24 (92)	13 (72)	37 (84)		
Yes	2 (8)	5 (28)	7 (16)		

Variables marked with an asterisk () were tested using Fisher’s exact test rather than the chi-square test, due to low expected frequencies*.

A chi-square analysis was performed to evaluate the association between HPV status and various demographic, lifestyle, and clinical factors among ESCC patients (**Table 4**). The results showed no significant associations between HPV status and gender, age group, race, staple diet, vegetable consumption, hot drink intake, co-morbidities, family history of cancer, alcohol consumption, smoking, or combined alcohol and smoking use. Due to small expected counts, Fisher’s exact test was applied for race. To control for multiple comparisons, p-values were adjusted using the Benjamini-Hochberg false discovery rate (FDR) procedure. The raw p-values were consistently large, resulting in uniformly high adjusted p-values of 0.842 for all variables except race, which had a raw and adjusted p-value of 1.000. Given this uniformity and lack of statistical significance, adjusted p-values are not presented in **Table 4** but are reported here for transparency.

**Table 4:** Chi-square analysis of demographic, lifestyle, and clinical factors by HPV status in ESCC patients.

Variable	HPV status		Total	Raw p -value
	Negative	Positive		
	N=34	N=44	N=78	
Gender, n (%)				0.733
Male	18 (53)	25 (57)	43 (55)	
Female	16 (47)	19 (43)	35 (45)	
Race, n (%)				1.000*
African	33 (97)	42 (95)	75 (96)	
Caucasian	1 (3)	1 (2)	2 (3)	
Mixed	0 (0)	1 (2)	1 (1)	
Age group, n (%)				0.662
≤50	5 (15)	5 (11)	10 (13)	
>50	29 (85)	39 (89)	68 (87)	
Staple diet, n (%)				0.447*
maize	29 (94)	39 (93)	68 (93)	
pasta	1 (3)	0 (0)	1 (1)	
rice	1 (3)	3 (7)	4 (5)	
Vegetables (often), n (%)				0.555
No	13 (38)	14 (32)	27 (35)	
Yes	21 (62)	30 (68)	51 (65)	
Hot drinks, n (%)				0.765
No	9 (26)	13 (30)	22 (28)	
Yes	25 (74)	31 (70)	56 (72)	
Cor-morbidities, n (%)				0.562
No	25 (74)	29 (67)	54 (70)	
Yes	9 (26)	14 (33)	23 (30)	
Cancer family history, n (%)				0.724
No	31 (91)	38 (86)	69 (88)	
Yes	3 (9)	6 (14)	9 (12)	
Alcohol, n (%)				0.640
No	20 (59)	23 (53)	43 (56)	
Yes	14 (41)	20 (47)	34 (44)	
Smoking, n (%)				0.366
No	22 (65)	24 (55)	46 (59)	
Yes	12 (35)	20 (45)	32 (41)	

Variables marked with an asterisk () were tested using Fisher’s exact test rather than the chi-square test, due to low expected frequencies. None of the raw p-values were statistically significant. All were adjusted using the Benjamini-Hochberg procedure (FDR 5%) and remained non-significant after adjustment*.

We further conducted both univariate logistic regression and Firth logistic regression to assess the association between HPV status and selected clinical risk factors among ESCC patients. Hot drink consumption was included in the univariate model due to its exploratory relevance but was excluded from the Firth logistic regression model to minimize model instability given the small sample size and limited variability in the data. The findings were consistent across models: age group, alcohol consumption, and smoking were not significantly associated with HPV status (all p > 0.05) (**Table 5**).

**Table 5:** Unadjusted and adjusted logistic regression results for factors associated with HPV status among patients with ESCC.

Variable	Unadjusted OR (95% CI)	P-value	Adjusted OR (95% CI)*	P-value
Age Group				
≤50 vs. >50	0.74 (0.20 - 2.81)	0.662	1.36 (0.38 - 4.84)	0.634
Alcohol consumption				
Yes vs. No	1.24 (0.50 - 3.08)	0.640	0.80 (0.22 - 3.07)	0.736
Smoking				
Yes vs. No	1.53 (0.61- 3.83)	0.367	1.85 (0.51 - 6.72)	0.352
Hot drink consumption				
Yes vs. No	0.86 (0.32 - 2.33)	0.765	–	–

*Adjusted estimates from Firth logistic regression.

Univariate logistic regression analysis identified several factors that were significantly associated with HIV status (**Table 6**). Age group analysis (≤50 vs. >50) revealed a significant association (OR = 6.88, p = 0.020), indicating that ESCC patients under 50 years are more likely to be HIV-positive than those over 50. Both alcohol consumption (OR = 3.30, p = 0.013) and smoking (OR = 4.23, p = 0.003) were significantly associated with HIV status. Similarly, HPV18 positivity was significantly associated with HIV status (OR = 6.67, p = 0.005), indicating a higher likelihood of HIV infection among ESCC patients with HPV18. Furthermore, combined alcohol consumption and smoking (OR = 3.19, p = 0.020) were significantly associated with HIV status, suggesting that ESCC patients who both drink alcohol and smoke have an increased likelihood of being HIV-positive. In contrast, HPV16 and hot drink consumption were not significantly associated with HIV status (p > 0.05). We then fitted two multivariable Firth logistic regression models: a full model including all relevant predictors and a parsimonious model with four key variables (age, alcohol, smoking, and HPV18 status). In the full model, none of the associations remained statistically significant after adjustment (p > 0.05), although the effect of HPV18 positivity showed a potential trend (adjusted OR = 3.32, 95% CI: 0.79 - 13.93; p = 0.101).

**Table 6:** Unadjusted and adjusted logistic regression results for factors associated with HIV status among patients with ESCC.

Variable	Unadjusted OR (95% CI)	P-value	Adjusted OR (95% CI)*	P-value	Adjusted OR – parsimonious model† (95% CI)	P-value
HPV status						
Negative vs. Positive	1.14 (0.46 - 2.82)	0.776	–	–		–
Age category						
≤50 vs. >50	6.88 (1.35 - 34.97)	0.020	0.32 (0.07 - 1.61)	0.168	0.28 (0.06 - 1.37)	0.117
Gender						
Female vs. Male	0.68 (0.27 - 1.69)	0.406	2.58 (0.49 - 13.51)	0.261	–	–
Alcohol consumption						
Yes vs. No	3.30 (1.28 - 8.47)	0.013	2.56 (0.38 - 17.37)	0.337	1.71 (0.41 - 7.08)	0.459
Smoking						
Yes vs. No	4.23 (1.62 - 11.06)	0.003	6.34 (0.47 - 85.69)	0.164	2.59 (0.62 – 10.82)	0.191
HPV16 status						
Yes vs. No	0.38 (0.10 - 1.38)	0.140	–	–	–	–
HPV Not Detected vs. HPV16 Negative	0.59 (0.17 - 2.08)	0.414	–	–	–	–
HPV18 status						
Yes vs. No	6.67 (1.75 - 25.43)	0.005	3.32 (0.79 - 13.93)	0.101	4.17 (1.00 - 17.32)	0.050
HPV Not Detected vs. HPV18 Negative	2.63 (0.84 - 8.20)	0.095	2.06 (0.60 – 7.11)	0.251	2.30 (0.69 - 7.67)	0.177
Alcohol & smoking						
Yes vs. No	3.19 (1.21 - 8.37)	0.02	0.53 (0.03 - 10.73)	0.681	–	–
Hot drinks consumption						
Yes vs. No	1.08 (0.40 - 2.95)	0.875	–	–	–	–

In the parsimonious model, HPV18 positivity remained borderline significant (adjusted OR = 4.17, 95% CI: 1.00–16.97; p = 0.050. Age, alcohol, and smoking showed non-significant trends in the adjusted model.

### 3.3 Other risk factors of ESCC

None of the patients in our cohort had a history of achalasia or caustic injury. The most common staple diet was maize-based foods, with the majority of patients reporting regular vegetable consumption. Hot drink consumption was reported by 72% (n=56) of patients, all of whom drank tea or coffee at high temperatures, with tea being the most commonly consumed beverage. However, no statistically significant association was found between hot drink consumption and viral infections in our cohort. Only nine patients reported a family history of cancer, five of whom had suspected upper gastrointestinal cancers. However, the precise diagnosis was unknown in most cases, except for one patient who confirmed a first-degree relative with esophageal cancer. Among comorbidities, hypertension was the most prevalent, diagnosed in 16 patients (20.5%), followed by diabetes mellitus in five patients. Only two patients had both hypertension and diabetes. A chi-square analysis revealed a significant association between smoking and alcohol use among ESCC patients (p < 0.001); 88% of patients who reported smoking also reported alcohol consumption, compared to only 13% among those who do not smoke.

## 4 Discussion

Esophageal carcinoma is one of the most challenging cancers facing mankind globally, primarily because of poor prognosis associated with clinical presentation at an advanced disease state, which regrettably is the typical stage of presentation in the vast majority of patients (17). South Africa not only bears a considerable burden of this cancer, but also bears an enormous burden of carcinogenic viruses such as HIV and HPV (18, 19). Research has demonstrated that certain viral infections may have an influence on the development and progression of ESCC. For instance, HPV has been identified as a potential causative agent (20, 21), and people living with HIV have been reported to have a higher incidence of ESCC when compared to patients without HIV (14, 22).

HPV, which has been identified as the most frequent sexually transmitted virus worldwide (23), has been recognized as a key etiological agent in malignancies originating from squamous epithelium, such as in the cervical, anogenital, and oropharyngeal regions (24–27). Previous studies have shown that approximately 25% of head and neck cancers are caused by HPV and can be directly linked to oral sex exposure (28). The most commonly identified and reported HPV types related to carcinomas, are the high–risk strains HPV-16 and HPV-18. Both these strains have been recognized as category-1 carcinogenic agents in humans. They are also the two most often reported HPV strains related with malignancies of the upper aerodigestive tract. (29, 30). They were the two most commonly seen high-risk HPV strains linked to ESCC, cervical cancer, and oropharyngeal squamous cell cancers in previous studies (31–33). In this study, we found that HPV infection was common, with 56.4% of our ESCC population testing positive. Additionally, in line with earlier research, we discovered that HPV 16 and HPV 18 were the two most commonly observed strains. We found HPV 16 to be the most common, affecting 68.2% of HPV-positive patients and HPV 18 was present in 40.9% of HPV positives. Notable though, it is prudent to mention that previous studies assessing the prevalence of HPV in esophageal cancer detected much higher prevalence rates and wider range of HPV strains from their tumor; for example, a previous study from Pretoria found HPV infection in 90% of ESCC patients, and identified the most common genotypes as HPV 51 (62.0%), HPV 70 (48.0%), with HPV-16 coming in at 3rd position at 44.0% followed by HPV 82 at 34.0% (34).

A systemic review by Ludmir et al. found that the rate of HPV infection in ESCC exhibited notable geographic variation which correlated with ESCC prevalence. Regions with higher ESCC incidence rates showed higher HPV infection rates. However, the review did not demonstrate a significant oncogenic role of HPV in ESCC (35). Another systemic review also demonstrated significant geographic and inter-regional variation in the detection of HPV infection in ESCC (36). For example, an Australian study detected HPV in only 8/222 (3.6%) of archived FFPE blocks of esophageal SCC (with the most common strain being HPV 16 followed by HPV 35) (37); while another study from East Africa detected HPV in 63/118 (53.3%) of their ESCC FFPE blocks (also HPV 16 was the most prevalent) (38). South Africa as well shows notable geographic variations in the incidence rates of ESCC, with the eastern provinces (i.e., Kwazulu-Natal and Eastern Cape) exhibiting significantly higher incidence rates compared to other provinces (12, 39). For example, this study was carried out in the central highveld region and only identified just above 78 patients over a 36-month period, whereas another study in KwaZulu-Natal reported on 159 patients seen within a period of 15 months, equating to approximately twice our numbers in less than half the time (40). Another study conducted in the Western Cape province of South Africa showed a low incidence of ESCC, detecting only 114 patients over a 4-year period, and furthermore the study found that only 9% of that cohort was infected with HPV, with HPV 18 being the most common strain (41). As previously mentioned a study from Pretoria detected HPV in 90% of their patients (34). The current investigation discovered that 56.4% of ESCC patients were infected with HPV. However, the targeted nature of the HPV genotyping utilized in our investigation may also imply that other high-risk strains (such as HPV 35, HPV 51, and HPV 82) that were found in previous studies were present but were not tested for, thereby influencing the observed prevalence rate.

South Africa is faced with a high rate of HIV infections (13). HIV itself has been linked to increased risk of ESCC. A large retrospective study that compared esophageal and gastric cancer prevalence rates between 44,075 HIV-infected males and a matched non-HIV infected cohort found that the rate of ESCC was more than 2 times higher in HIV infected cancer patients compared to non-HIV infected individuals with incidence rates of 9.12 per 100,000 person-years versus 4.20 per 100,000 person-years, respectively, which translated to about a 58% higher risk of ESCC in the HIV infected group (14). Furthermore, HIV has been shown to increase the acquisition and progression of HPV related illnesses including HPV induced cancers (42, 43). For instance, women with HIV who have a persistent HPV infection were shown to have a six-fold increase in the risk of developing cervical cancer compared to those without HIV due to HIV-induced immunosuppression (44). Previous researchers have reported that the oral mucosa of HIV-positive individuals had a more pronounced prevalence of high-risk HPV strains when compared to HIV-negative individuals (45), with HIV infected individuals experiencing a 2–3 times higher prevalence rate of oral HPV when compared to non-HIV infected people (28). Moreover, in comparison with the general public, PLWHIV were found to be more likely to develop HPV-associated malignancies (46, 47).

This study did not find a statistically significant correlation between HIV and HPV in the overall ESCC cohort (p-value = 0.78). In fact, the rate of HIV infection was slightly greater in patients who did not have HPV than in those who did, at 44% versus 41%, respectively. Furthermore, 67% of individuals with HIV who were co-infected with HPV had the HPV-18 strain, revealing a borderline association between the HIV and HPV-18 (with Raw χ² p-value = 0.015 and BH adjusted p-value = 0.051). However due to the small sample size and absence of non-cancer control group the significance of HPV 18 in HIV positive patients needs further evaluation in larger studies for adequate interpretation.

Several risk factors are associated with ESCC including alcohol use and smoking (8). In a recent meta-analysis evaluating ESCC risk factors in the African ESCC corridor, smoking and alcohol were identified as significant risk factors. The percentage of ESCC attributable to tobacco use was 18%, while the percentage attributable to alcohol use was 12%, and the percentage attributable to both tobacco and alcohol use was 18% (48). Additionally, in comparison to HIV negative individuals, HIV-positive individuals were found to be more likely to engage in high-risk behaviors, such as alcohol consumption, smoking as well as risky sexual behavior (49, 50). In this current study univariate logistic regression suggested that HIV-positive patients were more likely to smoke and drink alcohol when compared to HIV-negative patients; with smoking reported by 61% versus 32% and alcohol use by 61% versus 27%, at p = 0.012 and p = 0.003, respectively. Combined alcohol and smoking use was also found more frequently in HIV-positive individuals (52% vs. 25%) with p = 0.017. A significant proportion of ESCC patients reported smoking and alcohol usage, either alone or in combination, regardless of their viral status (p < 0.001), with 88% of people who smoke reporting using alcohol regularly. However, when multivariate logistic regression and BH procedure adjustment (p>0.05) was conducted only smoking remained statistically significantly associated with HIV (p = 0.045). Therefore our study demonstrates a link between HIV infection and smoking in OSCC. Univariate and Firth logistic regression analysis evaluating the associations between HPV and demographics, lifestyle plus risk factors, found no statistically significant associations between HPV status and gender, age group, race, staple diet, vegetable consumption, hot drink intake, presence of co-morbidities, family history of cancer, drinking alcohol, smoking, or combined alcohol and tobacco use. It seems more research is still required to determine the incidence rate and influence of HPV infections in ESCC in SA.

Regarding age and HIV, a prior study conducted in South Africa discovered that patients with HIV were 10 years younger than the general population when they were diagnosed with ESCC (40). Similarly, univariate logistic regression in current study showed that ESCC patients under the age of 50 years were more likely to be HIV positive than those over 50yrs with p = 0.02. Numerous studies have linked consumption of hot beverages to the etiology of ESCC, with the purported mechanism being the recurrent thermal injury to the esophagus mucosa (51–55). Our study demonstrated that hot drink consumption was common in our population being reported by 72% (n=56) of our patient group. Almost all of them characterized their drinks as hot during consumption and the number of cups drank per day ranged between 2-4. Nonetheless, our study did not show any statistically significant association between hot drink consumption and viral infections in ESCC.

## 5 Limitations

Our study’s has several limitations including a small sample size (n=78), and the fact that it was conducted in a single tertiary center. The small sample size limits the ability to confidently extrapolate the findings of the study (56), while the single center focus implies that our findings should be treated with caution until further external validation from multicenter studies (57). Another limitation is the lack of additional clinical data about the severity of HIV disease such as the viral loads, CD4 counts and duration of ART which were not measured in this study, therefore the influence of viral latency and persistence on ESCC cannot be adequately assessed.

Referral bias may have occurred in this study due to more advanced cases being referred to SBAH potentially inflating the observed prevalence of HIV and HPV co-infections. This bias may not accurately represent the situation in Gauteng. Additionally, selection bias may occur if individuals living with HIV are more likely to consent to study participation. Given that urban tertiary care populations often exhibit higher HIV prevalence than national averages, findings from such cohorts may not be generalizable. These biases underscore the need for cautious interpretation of prevalence estimates and risk factor associations in studies conducted in specialized healthcare settings.

Furthermore, it is probable that the overall number of HPV positives was decreased by focusing on the most commonly reported high-risk HPV strains (HPV 16 and 18), rather than utilizing assays that tested a far wider variety of HPV strains. Using “wider-net kits” would most probably have yielded a larger number of HPV positive patients. The absence of detection for other oncogenic HPV types may not provide a complete understanding of the viral contribution to disease burden. Nonetheless, the clinical relevance of the other strains would still need to be determined. Thirdly, our study analyzed FFPE samples acquired from microscopic endoscopic biopsies, which have limits with regard to the quantity of tissues available (i.e., compared to resection specimens). In addition to this the use of FFPE samples can reduce PCR sensitivity due to DNA fragmentation and chemical modification, factors known to compromise nucleic acid integrity. This can occur despite the use of optimized extraction protocols and dedicated FFPE kits. As such fresh-frozen tissue samples would be the most desirable sample source for future studies.

## 6 Conclusion

Our investigation could not demonstrate a statistically significant association between HPV and HIV in esophageal squamous cell carcinoma patients at SBAH and findings show that HIV status may not have influenced HPV positivity rate between the study years, however the study was not sufficiently powered to detect any, if there was. The HPV 18 strain showed a slight trend towards predominance in esophageal tumors in HIV-positive people who also had HPV infection.

## 7 Future research

Larger multicenter studies are required to evaluate HIV and HPV co-infection in ESCC, particularly in high-risk regions. Ideally, these investigations should be conducted on fresh tissue samples that have been appropriately preserved rather than utilizing FFPE tissues. The HPV status of histologically proven normal tissues adjacent to the tumor, as well as epigenetic expression profiles of both tumor and adjacent normal tissue, should be evaluated. Further research into the significance and potential trend of HPV 18 in HIV positive patients being associated with ESCC, including the biological mechanism, underlying potential oncogenic synergy between HIV infection and HPV in esophageal carcinogenesis has to be undertaken. This would offer valuable insights into co-infection dynamics and cancer susceptibility in immunocompromised populations. This would also require an expanded scope of HPV genotyping to fully establish the involvement of HPV. Future studies may also identify molecular alterations beyond viral detection.

## Data Availability

The original contributions presented in the study are included in the article/supplementary material. Further inquiries can be directed to the corresponding authors.
